# First microscopic and molecular identification of *Cryptosporidium* spp. in fat sand rats (*Psammomys obesus*) in Egypt and their potential zoonotic implications

**DOI:** 10.3389/fvets.2024.1488508

**Published:** 2025-01-23

**Authors:** Sara Abdel-Aal Mohamed, Fatma A. S. Anwar, Ahmed Gareh, Marwa M. I. Ghallab, Remigio Martínez, Asmaa Aboelabbas Gouda, Fatemah Enad Alajmi, Hind Alzaylaee, Ignacio García-Bocanegra, Ehab Kotb Elmahallawy

**Affiliations:** ^1^Department of Parasitology, Faculty of Veterinary Medicine, Assiut University, Assiut, Egypt; ^2^Department of Zoology, Faculty of Science, Assiut University, Assiut, Egypt; ^3^Department of Parasitology, Faculty of Veterinary Medicine, Aswan University, Aswan, Egypt; ^4^Department of Medical Parasitology, Faculty of Medicine, Kafrelsheikh University, Kafr El Sheikh, Egypt; ^5^Departamento de Sanidad Animal, Grupo de Investigación en Sanidad Animal y Zoonosis (GISAZ), Universidad de Córdoba, Córdoba, Spain; ^6^Department of Parasitology, Faculty of Veterinary Medicine, Zagazig University, Zagazig, Egypt; ^7^Department of Biology, College of Science, University of Hafr Al Batin, Hafr Al Batin, Saudi Arabia; ^8^Department of Biology, College of Science, Princess Nourah bint Abdulrahman University, Riyadh, Saudi Arabia; ^9^CIBERINFEC, ISCIII CIBER de Enfermedades Infecciosas, Instituto de Salud Carlos III, Madrid, Spain; ^10^Department of Zoonoses, Faculty of Veterinary Medicine, Sohag University, Sohag, Egypt

**Keywords:** *Cryptosporidium parvum*, morphology, molecular identification, sand rat, Egypt

## Abstract

**Introduction:**

Rodents, thriving in human-altered environments, pose significant public health risks due to their role as reservoirs for numerous zoonotic parasites. Among these, *Cryptosporidium* spp. are recognized globally as leading causes of waterborne and foodborne diarrheal illnesses in humans. The specific role of fat sand rats (*Psammomys obesus*) in the transmission of *Cryptosporidium* spp. in Egypt and the genotypic characteristics of the circulating species in these animals remain poorly understood.

**Methods:**

In this study, a total of 150 individual fat sand rat stool samples were collected from the saline marsh periurban areas of Abu-Rawash, Giza, Egypt. The samples were initially screened for the presence of *Cryptosporidium* spp. using light and scanning electron microscopy to characterize the parasite’s oocysts. Furthermore, molecular identification and characterization of the parasite were carried out on selected microscopy-positive samples (*n* = 30) using conventional polymerase chain reaction (PCR) targeting the *Cryptosporidium* oocyst wall protein (COWP) gene. A subset of these positive samples by PCR was subjected to sequencing, with the resulting sequences deposited in GenBank™ and analyzed through phylogenetic methods.

**Results:**

Conventional microscopy revealed that 46.7% (70/150; 95% CI: 38.7–54.6) of the analyzed stool samples contained structures consistent with *Cryptosporidium* oocysts. Moreover, the molecular analysis confirmed *Cryptosporidium* species in DNA from all 30 stool samples previously identified as heavily infected through microscopy. Notably, the phylogenetic analysis identified *Cryptosporidium parvum* (*C. parvum*) in the sequenced samples, likely originating from the rats’ native habitats. These identified species have been deposited in GenBank™ under the accession numbers OM817461 (*C. parvum* FSA-1), OM817462 (*C. parvum* FSA-2), and OM817463 (*C. parvum* FSA-3) and revealed closed genetic identity with those species reported from human and other animal species in the same geographic location.

**Conclusion:**

Overall, this study represents the first morphological and genetic identification of *C. parvum* isolated from fecal samples of fat sand rats trapped from periurban areas in Egypt. These findings provide valuable insights into the potential zoonotic implications of rodents in disease transmission at the national level, offering crucial information for public health awareness campaigns and informing local authorities.

## 1 Introduction

*Cryptosporidium* spp., protozoan parasites from the phylum Apicomplexa, are significant contributors to the global incidence of diarrheal diseases in humans and a wide range of animal species (1, 2). These organisms are responsible for cryptosporidiosis, a disease with considerable zoonotic potential. The epidemiological profile of these foodborne and waterborne parasites encompasses a wide range of domestic and wild animals, in addition to humans. Transmission of the infection occurs through consumption of contaminated water, food, or vegetables, as well as through direct contact with infected animals or humans (3, 4). While some infections may be asymptomatic, symptomatic cases often experience prolonged symptoms such as moderate-to-severe diarrhoea, lose or watery stools, stomach cramps, gastrointestinal discomfort, and mild fever (5, 6). It should be stressed that the genus *Cryptosporidium* comprises multiple pathogenic species, among them *C. parvum*, which is predominantly associated with human infections. In 2016 alone, acute infections caused by *C. parvum* led to over 48,000 deaths globally, especially affecting children with diarrhoea (7).

Despite the urgent need, there are currently no commercially available vaccines to protect humans or animals against cryptosporidiosis (8). Effective control strategies require integrated approaches and a One Health perspective to address the interconnected health of humans, animals, and the environment. The conventional method for diagnosing the infection involves microscopically examining stained fecal samples. This approach is widely used due to its simplicity and cost-effectiveness (9). On the other hand, molecular methods, such as genotyping and subtyping, have higher sensitivity and aid in tracing infection sources, understanding pathogen circulation in specific populations and regions, and assessing potential zoonotic spillover (10–12).

Importantly, rodent-borne diseases pose significant global health challenges, often categorized as neglected tropical diseases (13, 14). The transmission of pathogens between rodents and humans is more likely in areas where they cohabit, such as agricultural regions, peri-urban zones, and densely populated urban environments, especially when rodent populations are abundant (14, 15). Urbanization and climate change further exacerbate the spread of diseases carried by rodents, including bacteria, viruses, and parasites. *Cryptosporidium* spp. are particularly prevalent among rodents, with studies documenting a diverse array of species and genotypes, totalling 14 species and over 57 genotypes (1, 14). Among these, *C. parvum* is well-known for infecting humans and other mammals, as well as for its extensive genetic diversity within rodent populations (16).

*Psammomys obesus* (*P. obesus*), also referred as the fat sand rat, is a diurnal rodent belonging to the family *Muridae* (17, 18). It is found in North Africa, ranging from Mauritania to Egypt and Sudan, and extends into the Arabian Peninsula (19). This species is commonly predominantly residing in sandy desert habitats, although it can also inhabit rocky areas or saline marshlands. It exhibits a highly selective diet, consuming only the stems and leaves of plants within the *Amaranthaceae* family (20). It is important to highlight that *P. obesus* is recognized as can act as reservoir host for numerous zoonotic diseases (14). However, based on existing literature, there is currently no available information regarding the potential role of sand rats in transmitting *Cryptosporidium* spp. in Egypt. Therefore, our aim was to investigate and identify *Cryptosporidium* spp. in stool samples from fat sand rats collected from saline marsh areas (Navigations) of the Abu-Rawash, Egypt.

## 2 Materials and methods

### 2.1 Ethics statement

The ethical approval was obtained from the Committee of the Faculty of Veterinary Medicine, Assiut University, Egypt (No. 06/ 2042/ 0160).

### 2.2 Rodent collections

**Figure 1:**
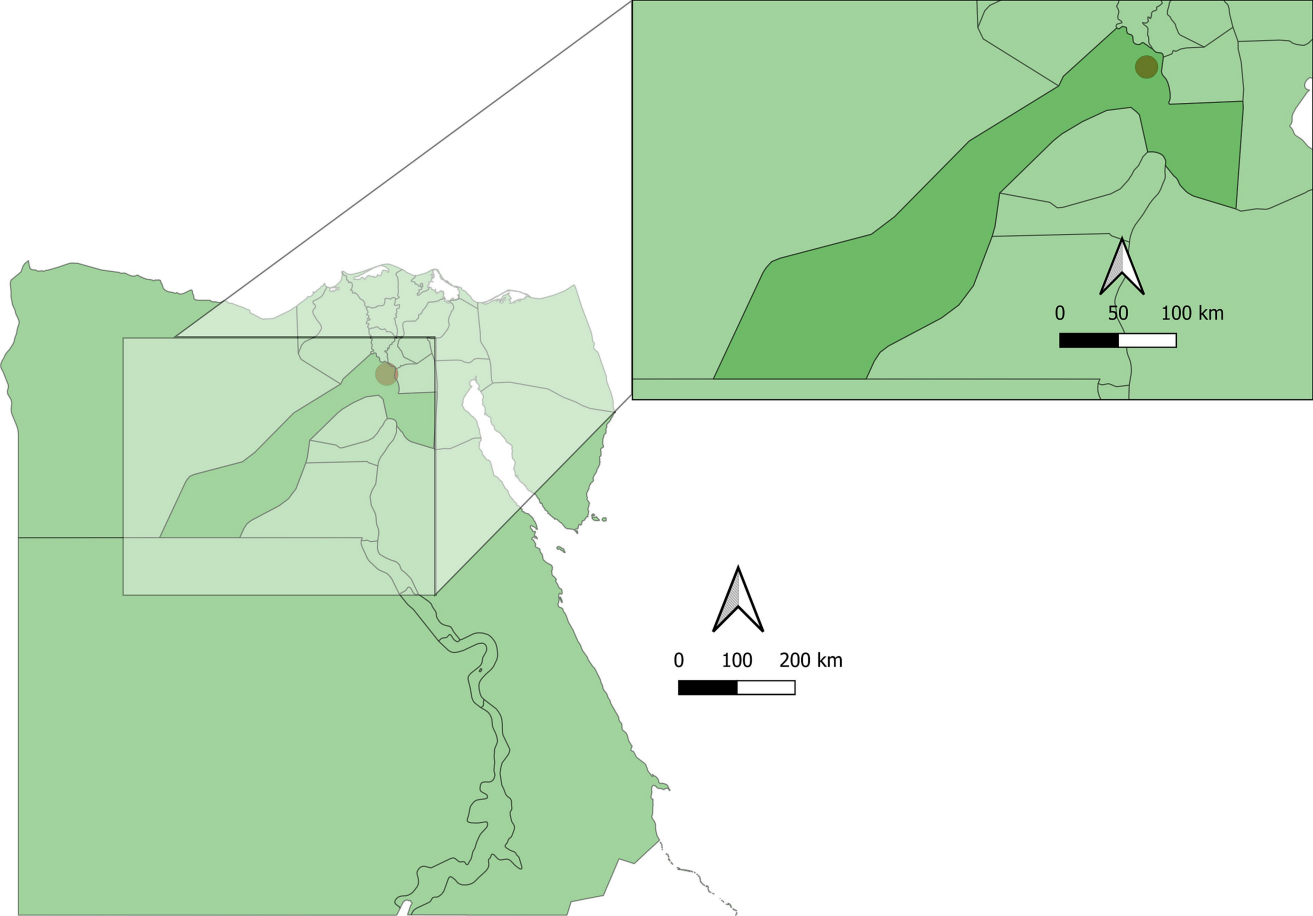
Map of the sampling sites and studied area. GIS files Copyright 2024 Pareto Software, LLC released under the creative commons by 4.0 license, https://simplemaps.com.

From December 2022 to November 2023, a total of 150 fecal samples were collected from fat sand rats in multiple saline marsh areas (Navigations) across 10 locations in the Abu Rawash area of Giza. Abu Rawash, situated in Giza Governorate, Egypt. This area is part of Egypt’s Western Desert, on the periphery of Greater Cairo. The area features a semi-arid climate with hot summers and mild winters. The map of the sampling studied area is shown in Figure 1. Sand rats were captured using 30 cage traps placed near burrow entrances as described elsewhere (19). These traps were baited with fresh sunflower seeds and either peanut or sesame butter, positioned at sunset, and retrieved early the following morning before sunrise. The captured rats were euthanized using CO_2_ inhalation as described elsewhere (21), and identified based on established criteria (22, 23), and then transported to the Parasitology Laboratory at Faculty of Veterinary Medicine, Assiut University (Egypt).

### 2.3 Parasitological investigation

For parasitological examination, fecal samples (approximately 500 mg) were collected directly from the intestine of each rat. Direct examination and sedimentation methods were employed, as previously described (24). Briefly, each sample was promptly processed and examined using routine direct fecal smear techniques with normal saline and Lugol’s iodine preparations. Additionally, fresh or preserved stool specimens (in 10% formalin) underwent concentration via centrifugal sedimentation in formalin-ether and formalin-ethyl acetate solutions, as well as centrifugal flotation in saturated Sheather’s sugar solution. Both the supernatant and sediment were fixed with methanol for 5 min before being smeared onto glass slides and stained using the modified Ziehl Neelsen technique. The samples stained with Ziehl Neelsen were then examined under light microscopy (1,000X magnification).

### 2.4 Scanning electron microscopy

For scanning electron microscopy, fecal samples are thoroughly mixed in a 10-mL saturated sugar solution (specific gravity 1.2) and then centrifuged at 1050 × g for 2 min (25). After centrifugation, 2 mL of the fluid from the meniscus, which contains the oocysts, is preserved in 70% alcohol until further use (26). *Cryptosporidium* sp. oocysts isolated from stool samples were then fixed in 5% glutaraldehyde in sodium cacodylate buffer for 1.5 h following the protocol described elsewhere (25, 26). They were then rinsed in distilled water and dehydrated in ethanol. After critical point drying, the samples were affixed onto stubs, coated with either carbon, then subsequently examined using a Joel JSM 35 Scanning Electron Microscope at 20 kV at Assiut University (Egypt).

### 2.5 Molecular detection

#### 2.5.1 DNA extraction and PCR amplification

Genomic DNA was extracted from 30 heavily infected stool samples, which had tested positive for the parasite via microscopic examination, using the QIAamp DNA Mini Kit (Qiagen, Valencia, CA, USA), according to the manufacturer’s instructions. A nested PCR technique was employed to amplify a specific fragment of the COWP gene following the protocols detailed in previous studies (27, 28). The initial PCR reaction targeted a 769-bp fragment of the COWP gene, using the following primer set: BCOWPF (5’-ACCGCTTCTCAACAACCATCTTGTCCTC-3′) and BCOWPR (5’-CGCACCTGTTCCCACTCAATGTAAACCC-3′). In the secondary reaction, a 553-bp fragment of the COWP gene was amplified using the primers COWP-Forward (5’-GGACTGAAATA CAGGCATTATCTTG-3′) and COWP-Reverse (5′-GTAGATAATGG AAGAGATTGTG-3′), as previously described (29). PCR amplification was carried out in a total volume of 25 μL and the reaction mixture comprised 2.5 μL of template DNA, 1× PCR buffer (Promega, Madison, WI, USA), 250 μM of each dNTP, 1.5 mM MgCl2, 10 pmoles of each primer, and 1.25 U of Taq DNA polymerase (28). The cycling conditions employed for the molecular identification of *Cryptosporidium* species in this study are provided in Supplementary Table 1. Subsequently, the resulting amplicons were electrophoresed on 1.5% agarose gels stained with RedSafe™ Nucleic Acid Staining solution and images were captured using a gel documentation system.

#### 2.5.2 Sequencing and phylogenetic analyses

PCR products from five positive samples, which displayed distinct and sharp bands, were purified using the QIAquick PCR Product Extraction Kit (Qiagen, Valencia, CA, USA). Following this, the sequence reaction was carried out using the Bigdye Terminator V3.1 cycle sequencing kit (Perkin-Elmer, Waltham, MA, USA), and subsequent purification was performed using Centrisep spin columns.

DNA sequences were then acquired using the Applied Biosystems 3,130 genetic analyzer manufactured (manufactured by Hitachi, Tokyo, Japan) and a Basic Local Alignment Search Tool (BLAST® analysis) (30) was conducted to determine sequence identity with GenBank^RM^ accessions. The final *C. parvum* sequences were named based on the primary subtype families identified through BLASTn searches, ensuring they exhibited >99% identity and adhered to the nomenclature method described elsewhere (31). These selected sequences of the COWP gene were deposited in GenBank via the National Center for Biotechnology Information (NCBI) under accession numbers OM817461 for *C. parvum* FSA-1, OM817462 for *C. parvum* FSA-2, and OM817463 for *C. parvum* FSA-3. To ensure a thorough comparison, sequences from various host species, including human samples from Egypt and other countries, were carefully selected. Alignment of these sequences was performed using ClustalW (32). A phylogenetic tree was constructed using the maximum likelihood method and the Tamura 3-parameter model (33), incorporating the bootstrap method with 1,000 replicates and MEGA Software (Version 11) was utilized for this analysis, selecting models based on the lowest Bayesian Information Criterion (BIC) (34).

## 3 Results

### 3.1 Frequency and morphological characteristics of *Cryptosporidium* spp. detected by microscopic methods

**Figure 2:**
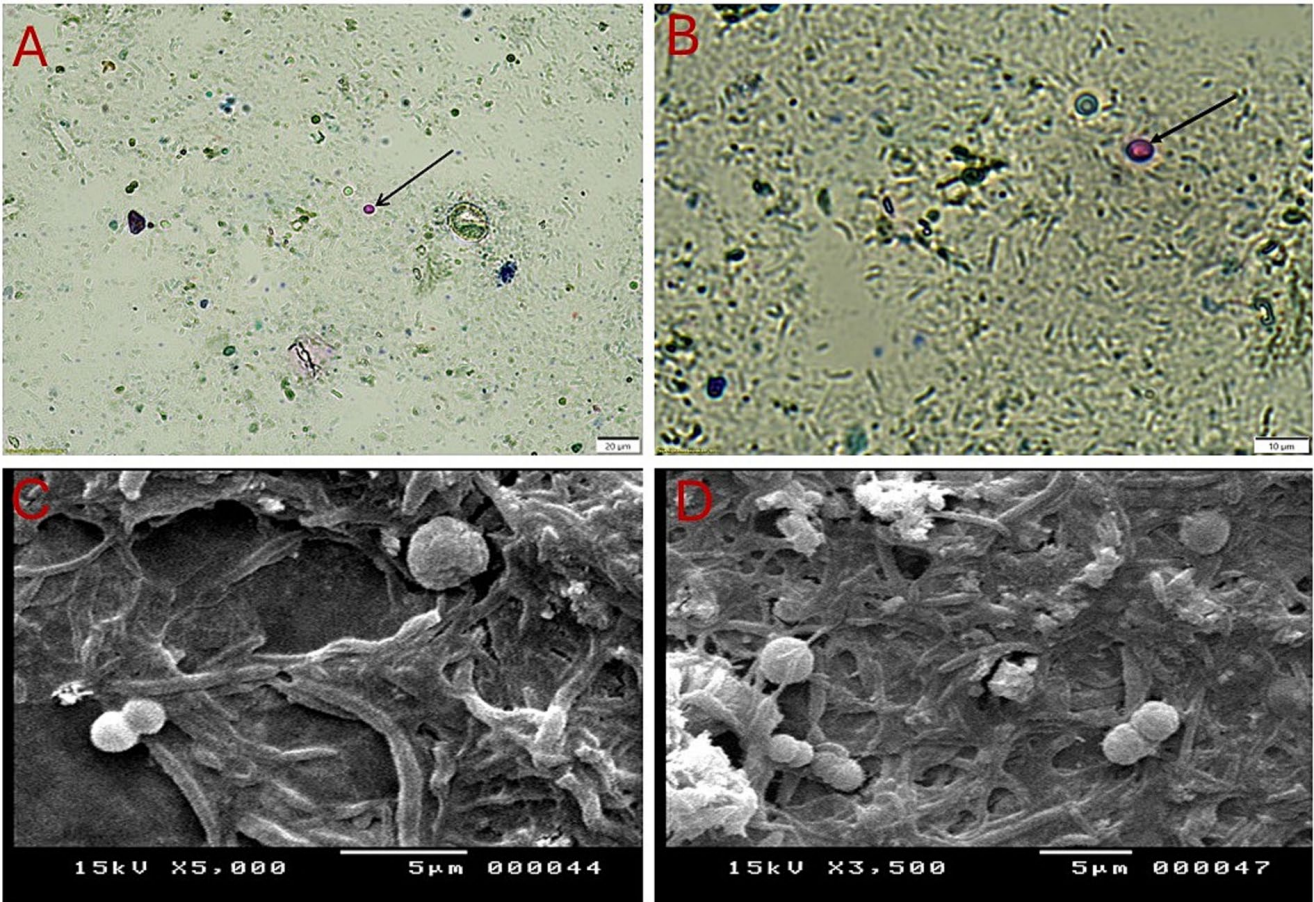
*Cryptosporidium* oocysts collected from the feces of sand rats (*Psammomys obesus*) captured using light and scanning electron microscopy. **(A,B)** *Cryptosporidium parvum* oocysts stained with Ziehl-Neelsen at different magnifications: **(A)** to be x100 and **(B)** to be x400. The oocysts appear spherical, stained red to pink, and are surrounded by a transparent halo. **(C,D)** Scanning electron micrographs (SEM) showing rounded oocysts.

In this study, microscopic examination of fecal samples collected from sand rats using centrifugal flotation method revealed that 46.7% (70/150, 95% CI: 38.68–54.65) of the samples had compatible forms with *Cryptosporidium* spp. Using direct methods and centrifugal sedimentation, *Cryptosporidium* spp. was detected in 20% (30/150, 95% CI: 13.60–26.40) and 26.7% (40/150, 95% CI: 19.59–33.74) of the tested samples, respectively. The oocysts detected exhibited uniform morphological features. As depicted in Figures 2A,B, microscopic analysis of fecal samples obtained from sand rats, stained with Ziehl-Neelsen dye, indicated the presence of *Cryptosporidium* spp. oocysts, which appeared spherical in shape and ranged in size from 4 to 6 μm. They also exhibited a characteristic red to pink coloration and are surrounded by a clear halo. The oocyst wall is notably thick, smooth, and colourless, lacking structures such as sporocysts, micropyles, and polar granules. Inner structures are minimally visible under a light microscope. When employing direct smears and the flotation method with Sheather’s solution, the oocysts of the parasite were observed as transparent circular structures with discernible walls (Figure 2). On the other hand, electron microscopy revealed that these oocysts are spherical cysts with thin, rough membranes of varying sizes (Figures 2C,D).

### 3.2 Genetic characteristics of *Cryptosporidium* species/subtypes

**Figure 3:**
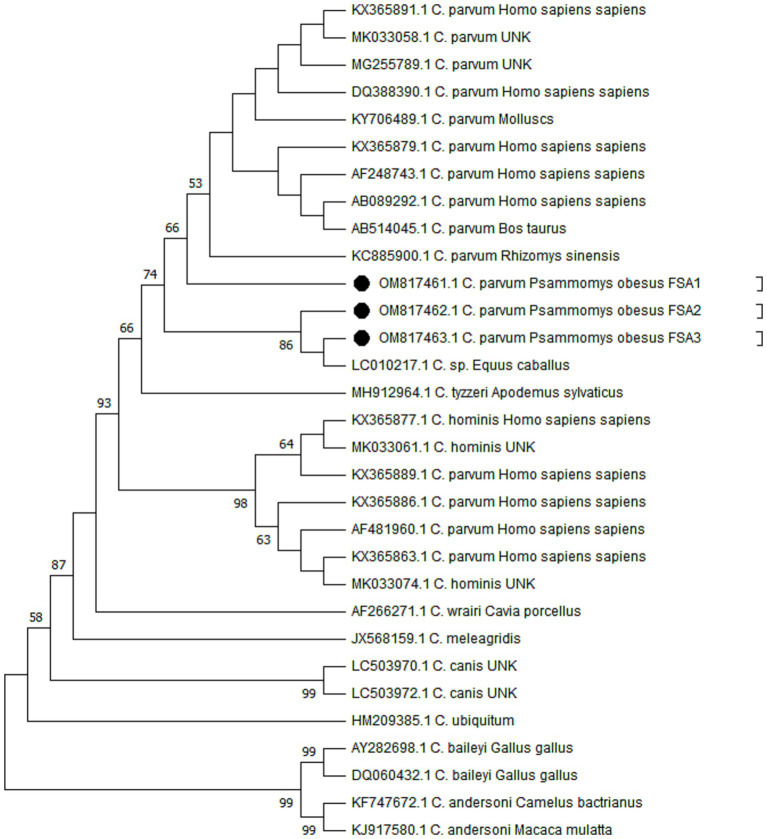
Phylogenetic trees constructed by the Maximum Likelihood method and Tamura 3-parameter model. The bootstrap consensus tree was inferred from 1,000 replicates and used to represent the evolutionary history of the taxa analyzed. The percentage of replicate trees in which the associated taxa clustered together in the bootstrap test (1,000 replicates) is shown next to the branches when values were larger than 50. The tree with the highest log likelihood (−1825.56) is shown. Initial tree(s) for the heuristic search were obtained automatically by applying Neighbor-Join and BioNJ algorithms to a matrix of pairwise distances estimated using the Tamura 3 parameter model, and then selecting the topology with superior log likelihood value. A discrete Gamma distribution was used to model evolutionary rate differences among sites (5 categories) (+G, parameter = 0.5552). This analysis involved 31 nucleotide sequences with a total of 478 positions in the final dataset. Evolutionary analyses were conducted in MEGA11. The sequence retrieved from the positive sand rats is given in black circles (accession numbers OM817461, OM817462, and OM817463).

Molecular analysis verified the presence of *Cryptosporidium* species in DNA extracted from 30 stool samples that had been identified as heavily infected based on microscopic examination. In this study, a total of five PCR-positive samples were subjected for sequencing. However, due to insufficient DNA, two *Cryptosporidium* spp.-positive samples were excluded from the genetic analyses. The phylogenetic analysis confirmed the presence of *C. parvum* (*n* = 3) among the studied samples (Figure 3). The sequences generated in this research have been submitted to the GenBank database under the accession numbers OM817461 for *C. parvum* FSA-1, OM817462 for *C. parvum* FSA-2, and OM817463 for *C. parvum* FSA-3. Figure 3 presents the Neighbor-Joining analysis comparing various *Cryptosporidium* species/genotypes alongside the isolated from sand rats in Egypt.

## 4 Discussion

Wild rodents play a crucial role in transmitting zoonotic pathogens within their ecosystems, highlighting their significant public health implications (35, 36). Their prolific reproductive capacity and frequent interaction with humans facilitate the spread of various pathogens through faecal shedding. More importantly, their close association with human habitats in urban, rural, and natural environments increases the risk of zoonotic pathogen transmission through contaminated food, water, and environmental surfaces (37). Therefore, understanding these dynamics is essential for implementing targeted disease surveillance and management measures to mitigate the risks associated with zoonotic diseases transmitted by wild rodents.

This present study provides novel insights into the role of sand rats in transmitting *Cryptosporidium* spp. and underscores their potential zoonotic implications in saline marsh and periurban areas in Giza, Egypt. Using a combination of microscopic techniques, our study revealed that 46.7% of the analysed fecal samples from rodents tested positive for *Cryptosporidium* spp., which represents a significantly higher detection rates compared to previous global studies. For instance, Zhao et al. (38) reported a lower prevalence of 28.6% in brown rats in Hainan Province (China) using molecular techniques, while Li et al. (39) found a prevalence of 12.2% in bamboo rats in another study in Guangdong Province (China) using PCR and sequence analyses of the small subunit rRNA gene. In another study in the Canary Islands (Spain), García-Livia et al. (40) documented prevalence rates of 13.9 and 10.3% among *Rattus rattus* and *Mus musculus domesticus*, respectively, using molecular methods. On the other hand, close results were recorded in a previous study in El Hierro, Canary Islands (Spain), with an overall prevalence of 48.6% for *C. parvum* of among murine species studied, *Rattus rattus* and *Mus musculus domesticus* (41). Given that the current data were derived from direct observation of oocysts in stool specimens using microscopic methods, which are routine but known for their low sensitivity, specificity and reliance on expertise (9), this fluctuation across studies can also be attributed to several factors that include the diversity of host species, abundance of rodents in studied area, sample sizes, geographical variations, climatic conditions and environmental influences, effectiveness of rodent control measures, hygiene practices, rodents’ access to human habitats and other animals, as well as variations in the diagnostic methodologies employed (39–43).

It should be noted that PCR is an increasingly preferred molecular method for detecting *Cryptosporidium* species in fecal samples due to its superior sensitivity and specificity over traditional microscopy (44, 45). It is therefore evident that PCR-based techniques with high sensitivity, specificity, and rapidly features can differentiate among species and genotypes in different specimens, including water, stool, and animal or human tissues (43). Adopting these molecular methods is essential not only to validate or confirm microscopic findings but also to identify circulating genotypes and species of the parasite. This approach might be crucial for gaining insights into the potential zoonotic transmission of the disease from rodents to humans. As depicted, the molecular analysis of examined stool samples revealed *C. parvum* based on the amplification of *Cryptosporidium COWP* gene. The BLAST analysis deposited the identified genotype under accession numbers: (OM817461 *C. parvum* FSA-1, OM817462 *C. parvum* FSA-2, andOM 817,463 *C. parvum* FSA-3). Similarly, a previous investigation (39) in China reported a *Cryptosporidium* bamboo rat genotype I, *C. parvum*, besides *Cryptosporidium* bamboo rat genotype III, *C. occultus*, and *C. muris*, whereas another study (42) documented *C. ditrichi* in *Apodemus flavicollis* (yellow-necked mouse) and *C. andersoni* in *Craseomys rufocanus* (grey-sided vole). Nonetheless, another study (38) in China recognized *Cryptosporidium* rat genotype I and IV, *Cryptosporidium suis*-like genotype and *C. ubiquitum*. In a prior study (40) conducted in the Canary Islands (Spain), *C. tyzzeri, C. meleagridis, C. muris* and *Cryptosporidium* sp. rat genotype I and II/III were identified among the examined rodents. More interestingly, the present study revealed a close genetic resemblance between the identified *C. parvum* parasites in rodents from this research and those previously recorded in GenBank, showing over 98% identity with human isolates from Egypt and other countries (46–49). This finding suggests that rodents may play a role in perpetuating this pathogen among humans.

The current study encounters some methodological limitations that might require consideration when interpreting the results. Primarily, due to financial constraints, the initial screening was based on light microscopy, and we were unable to conduct molecular analysis on all retrieved samples. Furthermore, COWP-based tools have demonstrated limited effectiveness in genotyping some *Cryptosporidium* spp. in animals due to their narrow specificity (50). Despite these limitations, our study represents the first molecular confirmation of *C. parvum* in sand rats in Egypt. These findings reflect that sand rats might act as reservoirs for *C. parvum* in this country. Given the zoonotic significance of *C. parvum* and the close genetic similarity between the sequences obtained and those found in humans in Egypt, the presence of rodents in human-inhabited areas—be they urban, rural, or natural—heightens the risk of pathogen transmission through contaminated food, water, and environmental surfaces. Further investigations are necessary to accurately ascertain the potential circulation of other *Cryptosporidium* spp. among rodents and other animals’ reservoirs cross the country.

## 5 Conclusion

This study provides novel preliminary insights into the potential role of sand rats in the transmission of *Cryptosporidium* spp. using a set of microscopic and molecular methods. Notably, it is the first molecular study to characterize *C. parvum* in sand rats (*Psammomys obesus*), which enhances our understanding for the potential zoonotic importance played by rodents in the transmission of cryptosporidiosis. Our findings emphasize the critical importance for public health education on the role of rodents in maintaining epidemiological foci of the pathogen and the necessity for enforcing strict hygienic measures by local authorities to eradicate rodents and prevent their access to human habitats. Conducting larger-scale, molecular-based epidemiological surveys will allow us to examine the prevalence and genetic characteristics of *Cryptosporidium* across diverse animal species and environmental samples. These studies will be vital in identifying infection sources, elucidating transmission pathways, and reducing the risk of disease spread from sand rats to humans.

## Data Availability

The original contributions presented in the study are included in the article/Supplementary material, further inquiries can be directed to the corresponding author.
