# Supplementary material for: First microscopic and molecular identification of Cryptosporidium spp. in fat sand rats (Psammomys obesus) in Egypt and their potential zoonotic implications
Source: Front Vet Sci. 2025 Jan 23;11:1488508. doi: 10.3389/fvets.2024.1488508 (PMC11800300; doi:10.3389/fvets.2024.1488508)
Supplement: Supplementary file 1 [file Table_1.DOCX]

**Supplementary Table 1.** PCR cycling conditions used for the molecular identification and/or characterization of the *Cryptosporidium* species and genotypes investigated in this study.

|  | **Temperature and time** | | | | | |  | |  | |  | |
| --- | --- | --- | --- | --- | --- | --- | --- | --- | --- | --- | --- | --- |
| **Locus** | | **Initial denaturation** | **Denaturation** | **Annealing** | **Extension** | **No. cycles** | | **Final extension** | | **Reference** | |  |
| *COWP* (Primary) | | 95°C 3 min | 94°C 1 min | 65°C 1 min | 72°C 1 min | 30 | | 72°C 10 min | | (27-29) | |  |
| *COWP* (Secondary) re) | | 95°C 3 min | 94°C 50 s | 55°C 30 s | 72°C 1 min | 30 | | 72°C 10 min | |  | |  |
